# Malarial Hæmaturia—Answer to Dr. McHatton

**Published:** 1891-11

**Authors:** E. H. M. Parham

**Affiliations:** Fordyce, Ark.


### MALARIAL HÆMATURIA—ANSWER TO DR. McHATTON.

BY E. H. M. PARHAM, M. D., FORDYCE, ARK.

I am not aware that the above named disease is not a hemorrhage as it appears in our common country; notwithstanding Dr. McHatton speaks so confidently of its being a secretion or excretion as he may see fit to call it. While I do not deny, but that broken down blood corpuscles may be discharged through the kidneys, but I will here state without the fear of successful contradiction, that a large majority of cases coming under my care, were attended with hemorrhage proper, the discharge coagulating readily and having all of the characteristics of blood, discoverable without the aid of a microscope. I recollect very well reporting a case, which was published ten or twelve years ago in the Southern Medical Record, which was attended with frightful hemorrhage from the stomach, bowels, and kidneys, having also all of the other symptoms commonly attending the disease under consideration. This case recovered without the use of a single grain of quinine.

The Doctor seems disposed to deal in prevention medicine in the treatment of this disease, which is all very well as far as it goes, but surely we cannot treat a disease until it exists; and he says that he uses quinine to prevent the breaking down of the blood corpuscles, knowing that it has a controlling influence over the entire malarial group. Now I would be delighted if the Doctor would tell me what malaria essentially is and the modus operandi of quinine in exercising the controlling influence over it, of which he so confidently speaks; I confess that I do not know.

I deny that quinine has a controlling influence over continued fevers, whether caused by malaria or not, and claim that it is only when the fever intermits, or assumes a periodical type, that we can confidently rely on it; and I have always endeavored to cause that intermission by treating the symptoms, viz: to meet such indications as appear from time to time. I mean to correct morbid secretion, supress hemorrhage, etc., and not rely exclusively on the attempt to prevent the action of a supposed poison, such as malaria, a vague and indifferent term at best, and a name given to a thing known only by its effects, which effects I propose to treat.

In regard to the use of ergotole in the treatment of malarial hemateria, I did not say, and did not mean to be understood as having treated all the cases coming under my care with that particular preparation. I only recommended it, now, as a nice preparation, to be used in the place of the fluid extract, as reliable and more pleasant to take than other preparations of ergot.

The doctor says that I indulged in a little pleasantry at his use of the word evacuant. Certainly he must have been dreaming and replying to some one else, as I fail to find any word or sentence in my article, making any allusion to it whatever,—in fact, I do not know that the word evacuant is in my article at all.

I will here state, and let it go for what it is worth, that I have been treating cases of malarial hæmaturia of various degrees of violence for the last twenty years, having a few cases almost every year, and, I am sure, more than one hundred cases in all, and I have not lost but one, and that one was under treatment for organic heart disease when he was taken with fever, and I am sure that it would seem from the above statement that clinical experience was of incalculable benefit to my patients, especially as the first case I had was a frightful one, and I gave quinine a thorough trial and seeing that it aggravated every unpleasant symptom, I desisted from its use in time to save my patient. The doctor says that I do not care for statistics, and I readily admit that they and theory have but little influence with me when they conflict with facts established by clinical experience.

I am not alone when I advance the opinion that when the fever, called malarial hematuria, is fully established that quinine aids materially in breaking down blood corpulsces or hemorrhage, whichever it may be, as the discharge always increases in proportion to the amount of quinine used.

With all due respect for the opinion of Dr. McHatton, and without any disposition to engage in controversies of any kind, I offer this for publication, hoping that it may cause those who are disposed to rely on quinine as a curative remedy in the treatment of malarial hematuria, to do it with watchfulness and caution, and to desist from it as soon as they find out its impotency in curing this disease. If it should do that much I will feel fully compensated for the trouble I have been at in writing this article, and I am sure that those who will take this precaution will never regret it.

